# Clinical education in reducing contamination rate in blood culture collection

**DOI:** 10.1186/1753-6561-5-S6-P286

**Published:** 2011-06-29

**Authors:** ML Thong, N Ambigadevi, MZ Noor Zaitulakma, K Rusmawati, Y Nor Azura

**Affiliations:** 1 Laboratory & Blood Services, Kuala Lumpur, Malaysia; 2 Infection Control Unit, National Heart Institute, Kuala Lumpur, Malaysia

## Introduction / objectives

At the Institute, blood cultures are drawn by doctors. Since 2009, more than 5% of blood cultures grew organisms as contaminant and was a concern to the Infection Control Committee. Our target is to reduce to 3% to meet the standard set by the American Society of Microbiology. The objectives of the clinical education are to enforce best practices in blood culture collection and to use 2% chlorhexidine as a skin decontaminant.

## Methods

Our data revealed the highest contamination rates were from the pediatric wards. In August 2010, an education campaign was conducted on blood culture sampling techniques. We collaborated with the pediatricians and observed current practices. A hospital-wide Continuous Medical Education, “Towards Best Practices in Blood Culture” was conducted in October. The working group developed a training tool with visual protocol placed on blood collection trolleys.

## Results

We found adherence to proper technique of blood sampling and skin decontamination were not always followed. The collection of blood cultures were through indwelling catheters, as well as inadequate volume of blood collected and contact time of antiseptic were not observed. The clinical education on these challenges brought the rate down from 7% in August to less than 3% after the education. The rateÂ is less than 3% from October 2010 to March 2011.

## Conclusion

Clinical education of doctors play pivotal role in reducing contamination rate. It is imperative doctors are aware of proper cleansing of the site and adherence to the recommended techniques. The infection control team and the microbiology laboratory continue to monitor the contamination rate, conduct ward observations on collection procedures and feedback to sustain this initiative.

## Disclosure of interest

None declared.

